# Multimodal alterations of directed connectivity profiles in patients with attention-deficit/hyperactivity disorders

**DOI:** 10.1038/s41598-019-56398-8

**Published:** 2019-12-27

**Authors:** Muthuraman Muthuraman, Vera Moliadze, Lena Boecher, Julia Siemann, Christine M. Freitag, Sergiu Groppa, Michael Siniatchkin

**Affiliations:** 1 Department of Neurology, Movement Disorders and Neurostimulation, Biomedical Statistics and Multimodal Signal Processing, University Medical Center of the Johannes Gutenberg University Mainz, Mainz, Germany; 2 Institute of Medical Psychology and Medical Sociology, University Medical Center Schleswig Holstein, Kiel University, Kiel, Germany; 3 Department of Child and Adolescent Psychiatry, Psychosomatics and Psychotherapy, Autism Research and Intervention Center of Excellence, University Hospital Frankfurt am Main, Goethe University, Frankfurt am Main, Germany; 4 Department of Child and Adolescent Psychiatry and Psychotherapy Bethel, Ev. Hospital Bielefeld, Bielefeld, Germany

**Keywords:** Network models, ADHD

## Abstract

Functional and effective connectivity measures for tracking brain region interactions that have been investigated using both electroencephalography (EEG) and magnetoencephalography (MEG) bringing up new insights into clinical research. However, the differences between these connectivity methods, especially at the source level, have not yet been systematically studied. The dynamic characterization of coherent sources and temporal partial directed coherence, as measures of functional and effective connectivity, were applied to multimodal resting EEG and MEG data obtained from 11 young patients (mean age 13.2 ± 1.5 years) with attention-deficit/hyperactivity disorder (ADHD) and age-matched healthy subjects. Additionally, machine-learning algorithms were applied to the extracted connectivity features to identify biomarkers differentiating the two groups. An altered thalamo-cortical connectivity profile was attested in patients with ADHD who showed solely information outflow from cortical regions in comparison to healthy controls who exhibited bidirectional interregional connectivity in alpha, beta, and gamma frequency bands. We achieved an accuracy of 98% by combining features from all five studied frequency bands. Our findings suggest that both types of connectivity as extracted from EEG or MEG are sensitive methods to investigate neuronal network features in neuropsychiatric disorders. The connectivity features investigated here can be further tested as biomarkers of ADHD.

## Introduction

Functional imaging modalities such as electroencephalography (EEG) and magnetoencephalography (MEG) measure neural activity at a high temporal resolution. However, each of these modalities has advantages and drawbacks. MEG is superior to EEG in the identification of brain sources in cortical regions with dipoles of tangential orientation. Additionally, short-range connectivity can be better captured using MEG^1,2^. In contrast, EEG is characterized by higher sensitivity in localization of brain sources in cortical regions with dipoles of radial orientation and is better at assessing long-range connectivity^3–5^. However, EEG is prone to volume conduction effects, and this is less significant for MEG^6–8^. But there are still some measures of brain activity, which have not been investigated sufficiently in terms of possible advantages of EEG or MEG. This is especially true for connectivity measures on the source level.

In our previous study, we demonstrated functional and effective connectivity are sensitive to brain maturation on the source level attributed to brain oscillations for EEGs recorded at rest from healthy children and young adults^9^. The analysis of functional connectivity at the source level was performed using the dynamic imaging of coherent sources (DICS) tool^8,10–15^, the analysis of effective connectivity was carried out using renormalized partial directed coherence (RPDC)^16^. Additionally, to describe the time-resolved causality network we analysed temporal partial directed coherence (tPDC) which gives both the time and frequency dynamics of causal connections between different regions of the brain^10,17,18^. Although advantages and problems of EEG and MEG for DICS and RPDC have been in depth discussed before^7^, most differences between EEG and MEG have been tested in healthy subjects. It is not known whether both modalities bring up similar pathophysiological results in clinical populations and, which of the described features from DICS, RPDC, or tPDC, based on whether EEG or MEG would be able to optimally differentiate between patients and healthy controls. The design here is a “proof-of-concept” study to look for connectivity features which could differentiate between these two cohorts of subjects.

Attention deficit/hyperactivity disorder (ADHD) is a highly prevalent and disabling disorder characterized by inattentiveness, hyperactivity, and impulsiveness^19^. Among other mechanisms, abnormally distributed functional and effective connectivity variables have been repeatedly postulated in patients with ADHD^20,21^, introducing a new network perspective for the pathophysiology of this disorder^22,23^. To date, very few studies have addressed characteristics of the resting-state functional and effective connectivity based on brain oscillations in ADHD patients^24^. In healthy subjects, functional and effective resting-state connectivities associated with neuronal oscillations in EEG were identified as robust marker for developmental changes during maturation^9,25^. Because ADHD has been repeatedly discussed as a developmental disorder^23^, the hypothesis that patients with ADHD could be characterized by an abnormal connectivity pattern has been proposed but insufficiently tested till now^4^. Moreover, this question has not been investigated yet for directional networks fingerprints i.e. using DICS and RPDC methods nor have the associated EEG and MEG data been compared. Since ADHD patients are often characterized by performance variability, which may be related to instable network dynamics^26–28^, it should be tested whether the temporal dynamics of network connectivity is affected in patients in comparison to healthy controls. And finally, because in our previous study EEG outperformed MEG in DICS and RPDC measures on the source level^7^, we assumed that EEG would better differentiate between patients with ADHD and healthy subjects.

## Materials and Methods

The study was performed in accordance with the Declaration of Helsinki and was approved by the Ethics Committee of the Faculty of Medicine, Goethe University Frankfurt am Main, Germany. All participants as well as the parents/caregivers of the children gave written informed consent prior to participation.

### Subjects

Eleven male neurotypical controls (NTC) (mean age 13.1 ± 1.8 years, range 10–16) and 11 male children and adolescents with ADHD (mean age 13.2 ± 1.5 years, range 12–17) were included in this study. There were no age (t = 0.88; p = 0.44) or IQ (t = 2.34; p = 0.39) differences between both groups (t-test for independent samples). All subjects were right-handed according to the Edinburgh Handedness Inventory [29] and had normal or corrected-to-normal vision. All participants were mentally normally developed, had normal EEG, and were German native speakers. None of them took any additional medication or presented with any history of developmental disorders or language problems.

According to DSM IV-R^19^, all ADHD children met the criteria for combined type or hyperactive-impulsive type (314.01). The inclusion criteria were as follows: (I) ADHD without conduct disorders or tic disorders as diagnosed by an experienced child and adolescent psychiatrist; (II) No other neuropsychiatric as well as no documented comorbidities in a structured psychiatric interview ‘Kinder-DIPS’^29^; (III) Sufficient compliance of child and family; (IV) Normal school achievement; (V) IQ > 85 (based on the CFT-20R^30^ (ADHD patients IQ = 100.4 ± 8.85; NTC subjects IQ = 113.9 ± 14.56); and (VI) No MEG exclusion criteria (i.e. ferromagnetic body objects, or a history of claustrophobia). The diagnosis of ADHD was supported by the parents’ version of a German adaptive Diagnostic Checklist for ADHD (FBB-ADHD)^31,32^ and by the psychiatric interview ‘Kinder-DIPS’^29^. All ADHD children were on medication (methylphenidate) before inclusion into the study. Medication was stopped at least 48 h before recordings. Therefore, patients were medication-free throughout the whole recording.

### Data recording

In each subject, resting-state EEG-MEG recordings in an eyes-closed condition were acquired for five minutes in a fully relaxed state. MEG (275 sensor system, Omega 2005; VSM MedTech Ltd.) and EEG measurements (64-channel system, BrainProducts, Munich, Germany) were done simultaneously. For EEG, 56 channels were selected from 61 equidistantly placed scalp Ag–AgCl electrodes using a standard cap (FMS, Munich, Germany; extended international 10–20-system). MEG recordings were performed using a whole-head system at a sampling rate of 1200 Hz in a synthetic third-order gradiometer configuration. EEG recordings were also sampled with 1200 Hz. All data were filtered online with fourth-order Butterworth filters (300 Hz low pass; 0.1 Hz high pass). Before and after each epoch the subject’s head position relative to the gradiometer array was determined using three localization coils, one at the nasion and the other two located 1 cm anterior to the tragus of each ear on the nasion-tragus plane. The individual EEG electrode positions were digitized using the Polhemus system. Epochs with a head movement exceeding 5 mm were discarded from further MEG/EEG data analysis. For artefact detection, a horizontal and vertical electro-oculogram (EOG) was recorded via four electrodes; two were placed distal to the outer canthi of the left and right eye (horizontal eye movements), and the other two were placed above and below the right eye (vertical eye movements and blinks). The impedance of each electrode was measured with an electrode impedance meter (Astro-Med, Inc Grass Instrument Division, W. Warwick RI USA) and was kept below 15 kΩ. Data analysis was conducted off-line.

### EEG/MEG data pre-processing

Each recording was segmented into 1s-long high-quality epochs, discarding all data sections with visible artifacts (compare e.g^7^). Muscle artifacts were first removed using manual data inspection by an experienced neurophysiologist. Based on infomax independent component analysis (ICA)^33^ the ICA components were profiled by their topography, activation time course, and spectrogram were excluded from the back projection. All scalp channels were then transformed to the average reference^34^, and segments with remaining artifacts were removed. On average we were able to use for the analyses of functional and effective connectivity (EEG: 280 ± 4 ADHD; 280 ± 5 NTC; MEG: 278 ± 4 ADHD; 276 ± 6 NTC) epochs. The mean overall data length after these pre-processing steps did not differ between groups (*p* > 0.05, two-tailed paired t-test). Next, the power spectral density was estimated, whereby the EEG and MEG signals were parsed in 1 s windows. For each of these segments a fast Fourier transformation (FFT, Hanning window: 10%, frequency resolution of 0.25 Hz) was computed. The average band power was calculated as the integrated area under the absolute power spectrum in the specific frequency band of interest, divided by the width (in points) of the specific frequency band. For the spectral band source mean power and synchronization analysis (see below) across all sources, the following frequency bands were examined: delta (1–3 Hz), theta (4–7 Hz), alpha (8–13 Hz), beta (14–30 Hz), and gamma (30–49 Hz). The choice of frequency bands was based on our previous study in order to assure compatibility of results^9^.

### Source analysis

A full description of the coherence analysis is given elsewhere^9,15,35^. There are two major constraints in this analysis: First, the analysis is created on a single dipole model, which is not linearly correlated to other dipoles, and second, the signal-to-noise ratio is sufficiently high^11^. The fixed dipole model was used in which the dipole source responsible for the measured EEG potentials during an epoch remains at a constant location, the dipole moment vector maintains a constant orientation throughout the epoch, and only the magnitude varies. The output of the beamformer at a voxel in the brain can be defined as a weighted sum of the output of all EEG channels^36^. The weights determine the spatial filtering characteristics of the beamformer and are selected to increase sensitivity to signals from a voxel and reduce contributions of the signals from (noise) sources at different locations. The frequency components and their linear interaction are represented as a cross-spectral density (CSD) matrix. In order to visualize power in the brain at a given frequency range, a linear transformation is used based on a constrained optimization problem which acts as a spatial filter^36^. The spatial filter was applied to a large number of voxels covering the entire brain, assigning to each voxel a specific value of power. A voxel size of 5 mm was used in this study. The beamformer weights for a given source (at a location of interest) are determined by the data covariance matrix and the forward-solution (lead-field matrix — LFM). The LFM was estimated with specified models for the brain; in this study, a boundary element method^37^ with three layers (brain, skull, and skin) was applied. The volume conductor model was created using standard T1- magnetic resonance templates. We have used age-appropriate templates for developing the volume conductor model^38^. Part of the forward modeling and the source analysis was done using the open source software FieldTrip^39^. For both groups, the head was modeled using the individual electrode locations. The LFM contains the information about the geometry and the conductivity of the model. The complete description of the solution for the forward problem has been described previously^40^. The brain region peak voxels representing the strongest power in a specific frequency was identified in each individual. The peak power voxels were selected by a within-subject surrogate analysis to define the significance level, which was then used to identify areas in the brain as activated voxels for subsequent runs of the source analysis. In order to create tomography maps, a spatial filter using a voxel size of 5 mm was applied to all voxels (covering the entire brain). Once brain region peak voxels were identified, their activity in source space was extracted from the surface EEG. In a further analysis, all original source signals with several activated voxels were combined by estimating the second order spectra and computing a weighting scheme depending on the analyzed frequency range to form a pooled source signal estimate for each region as previously described^41,42^.

### Connectivity analyses

Coherence only reveals components that are mutually correlated between two signals in the frequency domain but does not provide the direction of information flow between signals. In contrast, RPDC is a technique based on the perspective of Granger causality (time domain) and performed in the frequency domain to detect causal influences (i.e., directed connectivity) in multivariate stochastic systems. We have used the pooled source signals from each source for the effective connectivity analyses. RPDC provides information on the direction of information flow between sources^16^. The multivariate model was based strictly on causality (i.e. not taking into account zero-lagged or instantaneous influences) and was used to model the pooled source signal estimates by an autoregressive process to obtain the coefficients of the signals in the defined frequency bands. The open source Matlab (The MathWorks, Inc., Natick, MA, USA) toolbox ARFIT (http://www.clidyn.ethz.ch/arfit/) was used to estimate the autoregressive coefficients from the spatially filtered source signals^43^. The correct model order required for the determination of these coefficients was estimated by minimizing the Akaike information criterion^44^. This criterion reflects a measure of the relative goodness of fit, which has the minimum loss of information of a resulting statistical model with an optimal order for the corresponding model^45^.

Using time-frequency causality we can not only focus on a particular frequency itself, but can also analyze the time-dynamics of the causality at that frequency. Based on state-space modelling, the time-frequency causality estimation method of tPDC relies on dual-extended Kalman filtering (DEKF)^35,46,47^. This results in an estimate of the time-varying dependent auto regressive (AR) coefficients: One EKF estimates the states and feeds this information to the second EKF, which estimates the model parameters and back propagates this information to the initial EKF. By concurrently using two Kalman filters working in parallel with one another, it is possible to estimate both states and model parameters of the system at each time instant. After estimating the time-varying multivariate (MVAR) coefficients, the next step is to use those coefficients for the calculation of causality between the time series. Since DEKF yields the time-varying MVAR coefficients at each time point, we can calculate PDC at each time point as well^46^. Afterwards, a time-frequency plot using all PDCs can be concatenated to produce a time-frequency plot. The precise distribution of the MVAR coefficients is not known; we used the surrogate method called bootstrapping^48^ to check for the significance of the results. This method is based on the random shuffling of the subjected time series and hence it is data-driven. In short, it divides the original time series into smaller non-overlapping windows and randomly shuffles the order of these windows to create a new time series. The MVAR model is fitted to this shuffled time series. This process is repeated 100 times and their average is calculated. The resulting value is the significance threshold value for all connections. This process is performed separately for each subject. The quality of EEG signals is prone to noise and suffers from volume conduction^49^, therefore effective connectivity measures such as RPDC and tPDC need to be tested for reliability^50^. In this context, some authors use the imagery part of coherence^49,51^ or time reversal technique (TRT)^9,50^. In a simulation study, Haufe and colleagues (2013)^50^ demonstrated that TRT can simultaneously limit the impact of undesired weak asymmetries (i.e. non-casual interactions caused by zero-lagged, instantaneous coherences (=volume conduction)) and preserve or even enhance the influence of strong asymmetries genuinely related to the underlying process (i.e. time- lagged causal interactions not caused by volume conduction). Hence, TRT was applied as a second significance test on the connections already identified by RPDC and tPDC using bootstrapping as a data-driven surrogate significance test. Accordingly, the RPDC and tPDC asymmetries should be insensitive to contributions from volume conduction or other instantaneous interactions. In addition, our RPDC and tPDC asymmetry calculation should completely revert by applying TRT, and therefore be only sensitive to strong causal interactions. We applied TRT on the RPDC and tPDC values on both groups (NTC and ADHD) during the 5-minute eyes-closed.

### Support vector machine classification

SVM is a powerful tool^52^ for nonlinear classification between two data sets. In short, the algorithm looks for an optimally separating threshold between the two data sets by maximizing the margin between classes’ closest points. The points lying on the boundaries are called support vectors, and the middle of the margin is the optimal separating threshold. In most cases the linear separator is not ideal so a projection into a higher-dimensional space is performed where the data points effectively become linearly separable. Here, we have used the 3^rd^ degree polynomial function kernel for this projection due to its good performance as discussed in^52^ and use the grid search (min = 1; max = 10) and examined 10 possible values and a heuristic value of gamma (0.25). The selection was checked by 10-fold cross validation by taking 90% of the data (9 groups) for training and 10% (1 group) for testing. The process of shuffling with the reallocation of the groups was repeated thrice to attain a robust average classification accuracy. For each tested parameter the average classification accuracy will be reported. A soft-margin classifier was used for every parameter, and misclassifications were weighted by a penalty constant C. In order to optimize classification accuracy this was calculated for every classifier. The validation scheme was used to assess whether the included parameters allow automated classification between groups. The vectors from the source power, source coherence, RPDC and tPDC were extracted and tested for the optimal parameter. The classification was conducted separately for each analysed parameter. In total we had 20 features namely four parameters (source power, source coherence, RPDC and tPDC) and at each of the five frequency bands. Finally, by including all the parameters (ALL) the accuracy was estimated. We recently applied the analyses to distinguish between two patient groups^53^.

### Statistical analysis

Frequency band-specific spectral source mean power differences were assessed by two-tailed t-tests. The significance of the sources was tested by a within-subject surrogate analysis, in which the surrogates were estimated by a Monte Carlo random permutation 100 times shuffling of one-second segments within each subject. The p-value for each of these 100 random permutations was estimated and then the 99th percentile p-value was taken as the significance level in each subject^15^. To ensure that none of the reported results (which are all calculated for pre-defined frequency bands) are confounded by group differences in individuals’ alpha frequency (IAF), we also estimated and compared individual band limits calculated as a percentage of the IAF^54^, as described in our previous study^9^. For the IAF, the mean (over EEG channels except for BCG, EOG) is clustered along the upper and lower boundaries of delta, theta, and beta. This is realized by taking 10% of the predefined band edges. An IAF of 10.1 Hz would thus have a lower (upper) delta band edge of 1.01 Hz (3.03 Hz), derived from 0.1 (10% of 1 Hz) * IAF (10.01 Hz) and 3.03 (10% of 3 Hz) * IAF (10.1 Hz). Following this, the grand averages per frequency band (over participants) were evaluated by comparing them with the defined frequency bands respectively. For this, we computed within-group (one-sample t-tests) and between-group comparisons (paired t-tests). After this, for the statistical comparison on the power, coherence, and RPDC values, the source power (first source), mean coherence (or interaction strength), mean RPDC, and mean tPDC values between all the sources were estimated for testing the significance between EEG and MEG using three ANOVA factors: Modalities (EEG vs. MEG), Frequency bands (delta, theta, alpha, beta, and gamma) and Groups (healthy subjects and ADHD patients). A Friedman two-way analysis of variance test was then performed to test for significant differences between these values. For all statistical analyses, the significance level was kept at p < 0.05 (corrected for multiple comparisons by using Bonferroni alpha adjustment).

### Ethical approval

The study was performed in accordance with the Declaration of Helsinki and was approved by the Ethics Committee of the Faculty of Medicine, Goethe University Frankfurt am Main, Germany. All participants as well as the parents/caregivers of the children gave written informed consent prior to participation.

## Results

### Analysis of source absolute mean power and coherence from EEG and MEG

**Figure 1:**
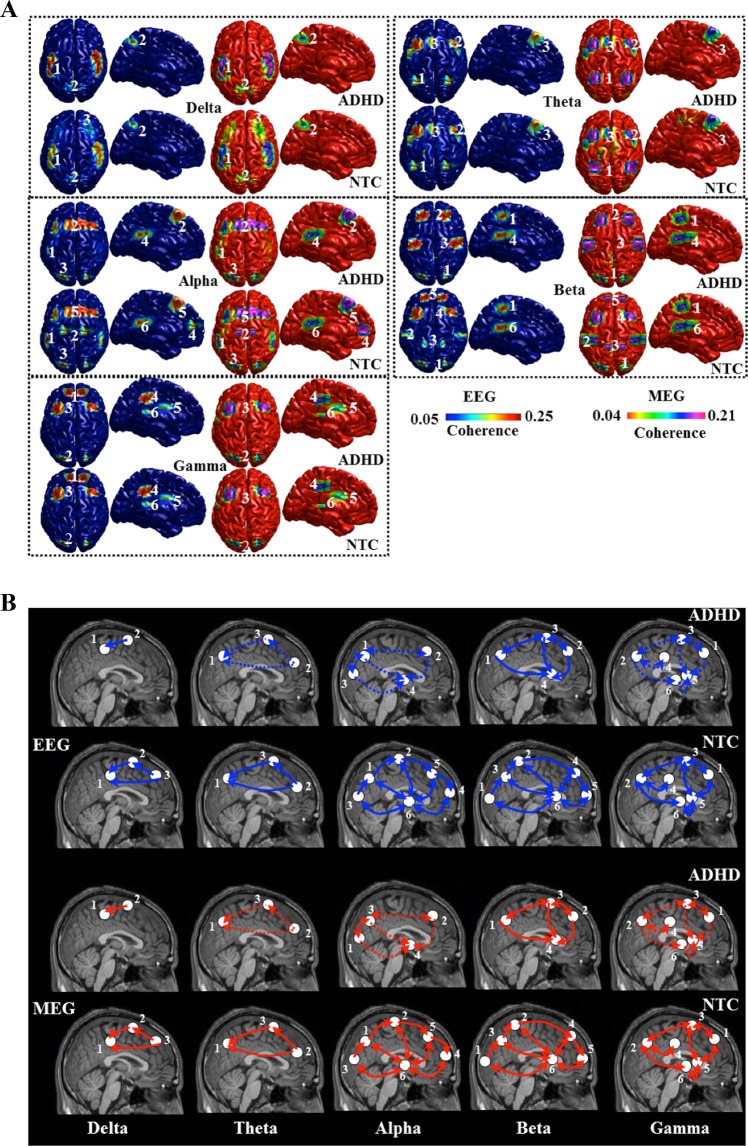
(**A**) Results of the frequency-band specific analysis of significant (p < 0.05) coherent sources. On the source level, healthy children showed stronger source mean power and coherence values in theta, alpha and gamma frequency bands compared to patients with ADHD. (**B**) Illustration of information flow between sources (same source naming as in (**A**) of brain activity for each frequency band. The directed within-group coherence analysis indicated significantly stronger information flow from frontal to parietal sources in control children for same frequency bands. In addition, thalamo-cortical connectivity was unidirectional (i.e., outflow from cortical regions) in ADHD group, but bidirectional in NTC group for the alpha, beta and gamma frequency bands.

The number of identified sources was lowest for the delta frequency band when compared to the other frequency bands (see Fig. 1A for an overview of the frequency band-specific coherence results). The sources for delta activity were present in the posterior cingulate cortex, and primary motor cortex in both groups. Additionally, inferior frontal gyrus was involved in the generation of delta activity in the control group. Theta band-related sources were located in the parietal cortex, medial prefrontal cortex, and dorsolateral prefrontal cortex in both groups. The number of sources involved in the alpha band was lower in ADHD group. Both groups of subjects revealed sources associated with alpha activity in the dorsolateral prefrontal cortex, premotor cortex, precuneus, and thalamus. However, in the NTC group additional sources were found in the visual cortex and motor cortex. For the beta frequency band, the number of sources was lower in the ADHD group and the sources were located in the posterior parietal cortex, primary motor cortex, inferior frontal gyrus, and thalamus. NTC subjects showed additional sources in the visual and medial prefrontal cortex. For gamma frequency band, the number of sources was comparable between NTC and ADHD. The sources were related to the dorsolateral prefrontal cortex, precuneus, primary motor cortex, Broca area, caudate nucleus, and putamen. All identified sources for the single frequency bands were comparable between both EEG and MEG. We estimated the Euclidean distance from the peak activated voxel for all the sources between the two groups for the frequency bands and did not find any significant differences for the location (p > 0.05). For the parameter source power we found significant main effect of Group F(1, 21) = 110.23; p = 0.009; and frequency band F(1, 21) = 80.92; p = 0.009; there was no significant interaction. The mean interaction strength we found significant main effect of Group F(1, 21) = 92.69; p = 0.008; and frequency band F(1, 21) = 71.39; p = 0.007; there was no significant interaction. For both recording modalities, the mean interaction strength between the sources for the frequency bands theta (ADHD > NTC: t = 4.58; p = 0.002; t = 3.87; p = 0.007), alpha (ADHD < NTC: t = 4.79; p = 0.001; t = 3.68; p = 0.006), and gamma (ADHD < NTC: t = 4.47; p = 0.003; t = 4.19; p = 0.005) were significantly different between the NTC subjects and ADHD patients. For the other frequency bands, there were no significant differences between the groups (see Tables 1 and 2**)**.

**Table 1:** Global EEG and MEG: first source (source power) differences between controls and ADHD.

Log power (Mean ± std)	ADHD (EEG)	NTC (EEG)	ADHD (MEG)	NTC (MEG)
Delta	4.26 ± 0.15	4.26 ± 0.14	4.07 ± 0.16	4.10 ± 0.11
Theta	1.75 ± 0.12*	1.24 ± 0.13	1.56 ± 0.15*	1.01 ± 0.14
Alpha	3.45 ± 0.65	3.74 ± 0.13	3.03 ± 1.04	3.64 ± 0.08*
Beta	0.17 ± 0.10	0.29 ± 0.18*	0.13 ± 0.10	0.17 ± 0.09
Gamma	0.31 ± 0.13	0.71 ± 0.16*	0.15 ± 0.07	0.58 ± 0.13*

*Represents p < 0.01 and **Represents p < 0.001.

**Table 2:** Global EEG and MEG: All sources (source coherence) differences between controls and ADHD.

Coherence (Mean ± std)	ADHD (EEG)	NTC (EEG)	ADHD (MEG)	NTC (MEG)
Delta	0.19 ± 0.03	0.19 ± 0.03	0.18 ± 0.04	0.16 ± 0.03
Theta	0.22 ± 0.03*	0.17 ± 0.05	0.18 ± 0.02*	0.14 ± 0.03
Alpha	0.11 ± 0.02	0.19 ± 0.02*	0.10 ± 0.02	0.18 ± 0.02*
Beta	0.13 ± 0.02	0.13 ± 0.03	0.13 ± 0.01	0.17 ± 0.09
Gamma	0.09 ± 0.02	0.14 ± 0.01*	0.08 ± 0.02	0.12 ± 0.01*

*Represents p < 0.01 and **Represents p < 0.001.

### RPDC-based directionality analysis from EEG and MEG

The information flow between sources (same nomenclature as in Fig. 1A) of brain activity for each frequency band is presented in Fig. 1B. One major finding of this study was that thalamo-cortical connectivity was unidirectional (i.e. outflow from cortical regions) in the ADHD group, but bidirectional in control children for the alpha, beta, and gamma frequency bands.

**Figure 2:**
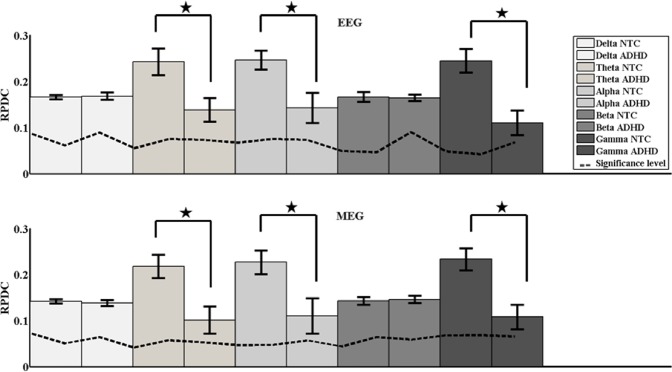
Shows the mean directed coherence for all the five frequency bands separately in bar (mean) and the error bars (standard deviation. The dashed line indicates the surrogate significance level and the asteriks (*) represents the significant differences between the two groups ADHD and NTC.

The RPDC analysis revealed generally lower and more unidirectional connections in the ADHD than in the NTC group (see Fig. 2). Additionally, in the ADHD group the connections between the cortical and subcortical sources were unidirectional, weak, and consisted of only top-down connections. In the NTC group we found bidirectional connections between cortical and subcortical sources. The strongest RPDC differences were evident for the theta, alpha, and gamma bands (see Table 3**)**. In comparison, it appears that for theta, ADHD patients showed lower RPDC values (RPDC maximum: 0.17 (EEG); 0.15 (MEG)) compared to the same unidirectional connections in NTC subjects (RPDC maximum: 0.28 (EEG), 0.26 (MEG)). For alpha, all the connections between the sources were weaker in ADHD patients and all the connections between the cortical sources (dorsolateral prefrontal cortex, pre motor cortex, precuneus) and thalamus were unidirectional from the cortical sources to thalamus. However, in NTC subjects the connections between the thalamus and the cortical sources were bidirectional and stronger. The connectivity patterns in the beta and gamma frequency bands were comparable to the alpha band, except that the connection strength in the beta frequency band was not significantly different between the NTC subjects and ADHD patients. All connections in the directed coherence were similar in both recording modalities.

**Table 3:** Global EEG and MEG: All significant directional connections (RPDC) differences between controls and ADHD.

RPDC (Mean ± std)	ADHD (EEG)	NTC (EEG)	ADHD (MEG)	NTC (MEG)
Delta	0.16 ± 0.01	0.16 ± 0.01	0.13 ± 0.02	0.14 ± 0.02
Theta	0.13 ± 0.02	0.24 ± 0.02*	0.10 ± 0.02	0.21 ± 0.02*
Alpha	0.14 ± 0.03	0.24 ± 0.02*	0.10 ± 0.02	0.22 ± 0.02*
Beta	0.16 ± 0.01	0.16 ± 0.01	0.14 ± 0.02	0.14 ± 0.02
Gamma	0.11 ± 0.02	0.24 ± 0.02*	0.10 ± 0.02	0.23 ± 0.02*

*Represents p < 0.01 and **Represents p < 0.001.

Based on statistical analyses, the directed coherence from the RPDC analyses we also found significant main effect of Group F(1, 21) = 83.17; p = 0.005; and frequency band F(1, 21) = 120.39; p = 0.004; there was no significant interaction. The mean directed coherence for theta (t = 4.24; *p* = 0.004 (EEG); t = 3.47; *p* = 0.008 (MEG)), alpha (t = 4.49; *p* = 0.002 (EEG); t = 3.92; *p* = 0.006 (MEG)), and gamma (t = 4.17; *p* = 0.003 (EEG); t = 4.06; *p* = 0.005 (MEG)) showed significant differences between groups (the directed coherence was stronger in NTC subjects compared to the ADHD patients). In contrast, there were no significant differences between groups concerning directed coherence for delta and beta frequency bands.

### Temporal partial directed source coherence (tPDC)

**Figure 3:**
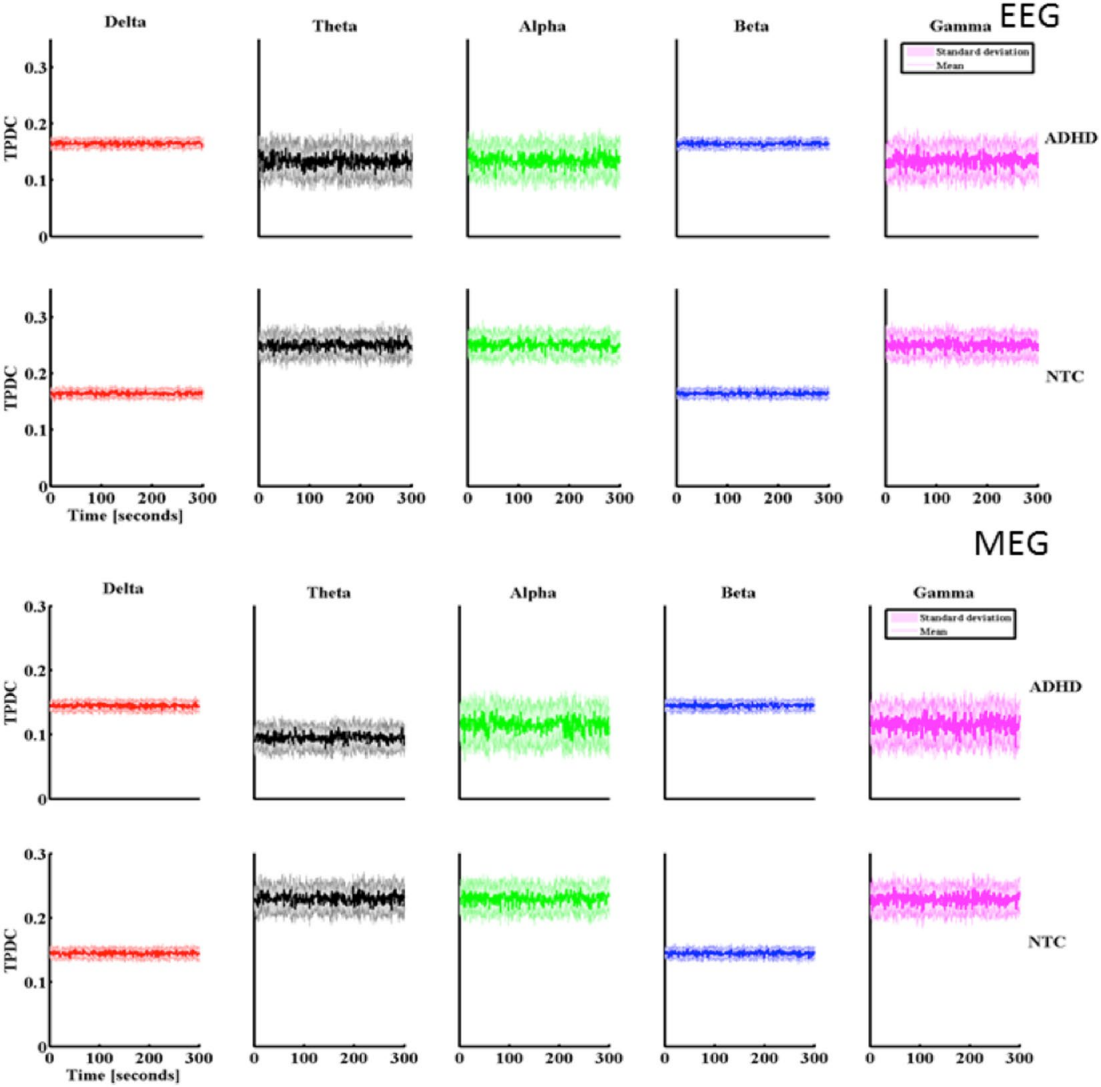
According to TPDC, ADHD group characterized significantly higher variance for theta, alpha and gamma frequency bands for both modalities EEG and MEG.

The tPDC analysis revealed higher variation in the connection strength over time for the frequency bands theta, alpha, and gamma but not delta and beta bands in both NTC subjects and ADHD patients. The directed coherence from the TPDC analyses we also found significant main effect of Group F(1, 21) = 1293.02; p < 0.001; and frequency band F(1, 21) = 1398.92; p < 0.001; however there was no significant interaction. There was a significant difference in the temporal dynamics (standard deviation) between the ADHD and NTC groups for theta (t = 2.24; p = 0.04; t = 2.57; p = 0.02, respectively), alpha (t = 2.31; p = 0.03; t = 2.65; p = 0.01), and gamma (t = 2.34; p = 0.03; t = 2.58; p = 0.02). The mean temporal connection strength was more variable over time in ADHD patients compared with NTC subjects in these three frequency bands (see Table 4). In contrast, there were no significant differences between the groups concerning tPDC for the delta and beta frequency bands. Moreover, there was no significant difference between recording modalities concerning tPDC as shown in Fig. 3.

**Table 4:** Global EEG and MEG: All significant directional connections (TPDC) differences between controls and ADHD.

TPDC (Mean ± std)	ADHD (EEG)	NTC (EEG)	ADHD (MEG)	NTC (MEG)
Delta	0.16 ± 0.03	0.16 ± 0.03	0.13 ± 0.03	0.14 ± 0.03
Theta	0.13 ± 0.07	0.24 ± 0.04**	0.10 ± 0.05	0.21 ± 0.04*
Alpha	0.14 ± 0.06	0.24 ± 0.03**	0.10 ± 0.07	0.22 ± 0.03**
Beta	0.16 ± 0.03	0.16 ± 0.03	0.14 ± 0.03	0.14 ± 0.03
Gamma	0.11 ± 0.06	0.24 ± 0.03**	0.10 ± 0.06	0.23 ± 0.03**

*Represents p < 0.01 and **Represents p < 0.001.

### SVM classifier on EEG and MEG

**Figure 4:**
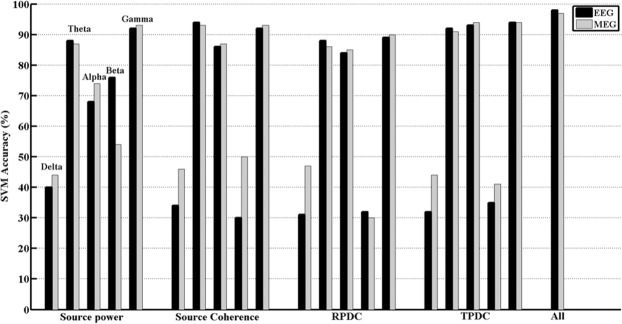
The features that showed accuracy above 90% are only discussed in the following section. The source power at gamma frequency showed (92%), source coherence at theta (93%) and gamma frequency band (92%), mean TPDC values at theta (92%), alpha (93%) and gamma (94%) and finally taking into all the features from the five different frequency bands showed (98%).

The classification accuracy differentiating ADHD and healthy groups for the analysed four connectivity measures is presented in Fig. 4. In case of source power, the maximal average classification accuracy was obtained for the gamma frequency band achieving 92.0% accuracy for EEG, 93.0% accuracy for MEG, followed by theta frequency band with 88% accuracy for EEG and 87% accuracy for MEG. For the source coherence, the maximum average classification accuracy was obtained for theta (EEG: 94% MEG: 93%) and gamma (EEG: 92% MEG: 93%), followed by alpha (EEG: 86% MEG: 87%) frequency bands. Concerning RPDC values, the best classification accuracy was found for gamma (EEG: 89% MEG: 90%), theta (EEG: 88% MEG: 86%) and then alpha (EEG: 84% MEG: 85%) frequency bands. In case of tPDC values, the highest average classification accuracy was described for gamma (EEG: 94% MEG: 94%), alpha (EEG: 93% MEG: 94%) and theta (EEG: 92% MEG: 91%) activities. Finally, using all the four parameters and including all five frequency bands, we achieved an average accuracy of 98% for EEG and 97% for MEG. The application SVM classifier highlighted the significance of theta, alpha and gamma oscillations in differentiation between ADHD and healthy groups.

## Discussion

Our goal was to examine functional and directed connectivity variables as derived from EEG and MEG in children with ADHD and compare them to neurotypical controls (NTC). Subsequently, to explore all these variables derived from several temporally resolved methods (beamformer source power, source coherence, RPDC and tPDC) for the quantification of the best biomarker for resting-state brain oscillations dynamics in ADHD. The key findings of this study are: (1) For all investigated features there were no substantial differences between EEG and MEG; (2) ADHD patients were characterized by a lower number of active sources than neurotypical control (NTC) subjects for the delta, alpha, and beta frequency bands; (3) Within each frequency band, the directed between-group coherence analysis indicated significantly stronger connectivity values from frontal to parietal sources in healthy children than in ADHD patients; (4) Thalamo-cortical connectivity was unidirectional (i.e. outflow from cortical regions) in the ADHD group, but bidirectional in the NTC group for the alpha, beta, and gamma frequency bands as derived from both EEG and MEG; (5) According to tPDC, the ADHD group was characterized by a significantly higher standard deviation for the theta, alpha,and gamma frequency bands. (6) The SVM results showed the theta, alpha and gamma oscillations are able to differentiate between ADHD and NTC with 98% for EEG and 97% for MEG separately. It seems likely that the chosen connectivity measures differentiate well between healthy children and patients with ADHD.

### Differences between EEG and MEG derived functional and effective connectivity values

In this study, we were able to demonstrate similar networks of functional (sources of activity as revealed by DICS) and effective (information flow between sources as described by RPDC) connectivity for the addressed frequency bands, confirming our previous studies on EEG data in healthy children, adolescents, and adults^9^, and on EEG and MEG data in healthy adults^7^. Therefore, it seems likely that the networks extracted from DICS and RPDC techniques are robust and stable. Moreover, the results of this type of network analysis were well replicable. In this work we focused on methodological aspects of the network analysis and refer to our previous publications^7,9,14,55^ for an extensive discussion about the described coherent networks found at each frequency bands. In addition to the robustness of our novel analysis approach of effective connectivity using tPDC, we found no differences between EEG and MEG recording modalities concerning measures of functional (DICS) and effective (RPDC and tPDC) connectivity on the source level.

While MEG measures brain activity with the tangential orientation of sources whereas EEG may characterize both radial and tangential activity of the brain, the methods may be differentially sensitive to measures of brain activity such as functional and effective connectivity. Indeed, it seems likely that the short-range connectivity could be better captured using MEG^56^ while EEG is more suitable in assessing long-range connectivity^3–5^. In our study, we investigated connectivity on the source level, finding no significant differences between modalities. These results agree with our previous comparisons between EEG and MEG (see^7^: The network of coherent sources and the spatial resolution were similar for both the EEG and MEG data when analyzed separately^7^. This is in line with other studies reporting no differences in connectivity measures between EEG and MEG^57^. Here, it may be concluded that both modalities - EEG (64 channel recording as used here) and MEG (275 sensor system)- may be used effectively for analysis of functional and effective connectivity with the aid of DICS, RPDC and tPDC methods for the quantification of wide band resting state processes. DICS, RPDC and tPDC analyses are moreover sensitive enough to differentiate between healthy subjects and patients with ADHD. This has important implications for clinical purposes: Since EEG is cost- and time-efficient and easily implemented, the nature of these abnormalities should be further characterized and translated into therapeutic interventions. Therefore, if DICS, RPDC and tPDC produce similar results for EEG and MEG, the method has a greater potential to be implemented in the clinics.

### Network differentiation between ADHD and NTC

EEG/MEG analyses present a powerful tool to shed light on the dynamic changes in neural network activities associated with ADHD (see e.g^27,58,59^). Apart from oscillatory instability, performance variability during tasks is a key finding in ADHD^26,28,60,61^. To date, a potential connection between the above findings has not been established. Here, we addressed this issue using diverse connectivity parameters based on EEG/MEG, including tPDC that is capable of depicting the dynamics of oscillatory abnormalities in ADHD. We found abnormalities in networks involved in focused attention, which potentially relates to concomitant performance fluctuations.

A major finding of the present study was the unidirectional nature of thalamo-cortical connectivity patterns in the alpha, beta, and gamma frequency bands (i.e. RPDC) in the ADHD group as compared with the bidirectional connections in control children. This directionality was characterized by an outflow from cortical regions to the thalamus and was weaker than in the control group. Cortical-subcortical connections are modulated during development (i.e. maturation), showing an increasing strength for the connections between thalamus and frontal cortex compared to a decrease from temporal cortex to thaalmus^62^. In ADHD, altered fMRI-based thalamic functional connectivity patterns were related to executive control deficits^63^. In addition, thalamo-cortical interactions putatively gate inhibitory control signals through alpha-specific EEG signals^64^. Thus, thalamic control signals correlate with increased alpha power that in turn predicted better task performance^65^. In particular, alpha-related phasic state changes from pre-stimulus to stimulus seem to create optimal conditions for focused attention^66^. In line with a potential inhibitory role for alpha oscillations during task, in the present study ADHD patients additionally demonstrated weaker coherence values as well as more variable network dynamics (as reconstructed from tPDC). Interestingly, the pattern in the theta frequency was analogous except for a stronger coherence (not weaker, as for alpha) than obtained in healthy controls (see^67^ for similar antagonistic dynamics between theta and alpha). It has been argued that alpha activity reflects arousal (i.e. “the current energetic level of the organism”) and that theta activities represent task- or situation-specific activation changes resulting from information processing^68^. Klimesch argued that low levels of theta activity and high levels of alpha activity during resting state predict increased theta power and decreased alpha power during task performance in healthy individuals, subsequently leading to improved cognitive performance^69^. Interestingly, ADHD seems to be associated with increased theta and decreased relative alpha activity^70^. More specifically, measures of theta and alpha are associated with numerous abnormalities in ADHD such as a disturbed shift from the default mode to the task mode (default mode interference^71^, developmental delays (maturation lag^72^), increased performance instability^73^, and hypo-arousal^74^). Potentially, these findings are interrelated, reflecting different symptoms of a common cause: The underlying mechanism may hence be the instable, non-optimal interplay between different neuronal oscillations, especially in the theta and alpha bands^75^. Thus, elevated theta power at rest has been associated with subsequent task-related information processing deficits in ADHD^76^, whereas reduced alpha activity was correlated with worse task performance^73^. The present findings thus contribute to a growing body of evidence suggesting that network instabilities, particularly of the theta and alpha frequencies, are key features of the ADHD pathology. Future research should focus on the mechanisms underlying neuronal as well as behavioral fluctuations to determine whether both are independent or whether they are directly or indirectly related to each other. A possible connection has been raised in the present study that requires further clarification, including a detailed characterization of the dynamics (e.g., trial-by-trial correlation) between oscillations and performance.

### Limitations of the study

There are some limitations when interpreting the results of this study. First, the small sample size may limit the generalizability of the present findings. However, the fact that we could show ADHD-specific patterns of functional and effective connectivity even in this small group speak in favour of the significance and are promising for future studies using the same methods in larger populations. As a second limitation, all the comparisons were performed with a 64-channel EEG system and a 275 sensor MEG system. In a previous study^7^, we analysed a comparable number of sensors in EEG and MEG recordings (64 sensors) and obtained no difference in the power and coherence measures between both modalities except that the spatial resolution was worse with less number of sensors for the MEG. The methods thus need to be applied to other systems in order to probe their generalizability. Additionally, in this study semi-realistic head models with individual electrodes and sensor locations were applied rather than a realistic head model. The advantages of using individual MRI for boundary element or finite element methods in localizing electrical sources are shown in previous studies^37,77^. Consequently, the sensitivity of the algorithms may increase when applying individual head models. Third limitation in this study, eventhough we have similar results for both the modalities separately, we have not combined both the modalities in our analyses, as they are complementary in nature. In our own previous work, we have shown this could be advantageous to combine both the modalities for connectivity analyses^7^ and to attain better spatial resolution^8^. Combining these modalities for source analyses might help in distinguishing between the primary and secondary interictal areas as described earlier in epilepsy patients^78^. Finally, the sub-cortical sources revealed in this study using DICS (i.e. the thalamus, putamen, caudate nucleus) are questionable, since, the identification of deep regions in the brain with scalp recordings is still under debate. Still, several studies using MEG^11,79^ and EEG^8,15,35,80^ have shown that the beamformer approach is a powerful tool for locating deep sub-cortical sources.

## Conclusion

This works is able to pinpoint the essential role of integrated analysis of brain oscillations at different frequency bands in differentiating a clinical group of young patients with ADHD from healthy controls. Methods of functional and effective connectivity based on both EEG and MEG data are in turn promising tools to generate robust biomarkers for ADHD pathology. Now, the results of the study have to be validated in a large clinical cohort.
